# GST-TAT-SOD: Cell Permeable Bifunctional Antioxidant Enzyme—A Potential Selective Radioprotector

**DOI:** 10.1155/2016/5935080

**Published:** 2016-05-30

**Authors:** Jianru Pan, Huocong He, Ying Su, Guangjin Zheng, Junxin Wu, Shutao Liu, Pingfan Rao

**Affiliations:** ^1^College of Biological Science and Engineering, Fuzhou University, No. 2 Xue Yuan Road, University Town, Fuzhou, Fujian 350108, China; ^2^Laboratory of Radiation Oncology and Radiobiology, Fujian Provincial Cancer Hospital, Teaching Hospital of Fujian Medical University, Fujian Key Laboratory of Tumor Translational Cancer Medicine, The National Clinical Key Specialty Construction Program of China, No. 420 Fuma Road, Fuzhou 350014, China; ^3^Food Nutrition Sciences Centre, Zhejiang Gongshang University, Room 407, No. 1 Laboratory Building, No. 149 Jiaogong Road, Xihu District, Hangzhou 310012, China

## Abstract

Superoxide dismutase (SOD) fusion of TAT was proved to be radioprotective in our previous work. On that basis, a bifunctional recombinant protein which was the fusion of glutathione S-transferase (GST), SOD, and TAT was constructed and named GST-TAT-SOD. Herein we report the investigation of the cytotoxicity, cell-penetrating activity, and in vitro radioprotective effect of GST-TAT-SOD compared with wild SOD, single-function recombinant protein SOD-TAT, and amifostine. We demonstrated that wild SOD had little radioprotective effect on irradiated L-02 and Hep G2 cells while amifostine was protective to both cell lines. SOD-TAT or GST-TAT-SOD pretreatment 3 h prior to radiation protects irradiated normal liver cells against radiation damage by eliminating intracellular excrescent superoxide, reducing cellular MDA level, enhancing cellular antioxidant ability and colony formation ability, and reducing apoptosis rate. Compared with SOD-TAT, GST-TAT-SOD was proved to have better protective effect on irradiated normal liver cells and minimal effect on irradiated hepatoma cells. Besides, GST-TAT-SOD was safe for normal cells and effectively transduced into different organs in mice, including the brain. The characteristics of this protein suggest that it may be a potential radioprotective agent in cancer therapy better than amifostine. Fusion of two antioxidant enzymes and cell-penetrating peptides is potentially valuable in the development of radioprotective agent.

## 1. Introduction

As a component of therapy for a wide range of malignant conditions, radiotherapy is estimated to be used by half of all cancer patients during the course of their treatment for cancer. The absorption of ionizing radiation by living cells can directly disrupt molecular structures, producing chemical and biological changes. Through radiolysis of water, it can also act indirectly by generating reactive chemical species that may damage nucleic acids, proteins, and lipids [1]. The direct and indirect effects of ionizing radiation initiate a series of biochemical and molecular signaling events [2]. Irradiation of noncancerous “normal” tissues during the course of therapeutic radiation can result in a range of side effects including self-limited acute toxicities, mild chronic symptoms, or severe organ dysfunction. To protect organisms from radiation, various agents, called radioprotectors, have been utilized. Amifostine is the only clinical radioprotector approved by the Food and Drug Administration (FDA) for head and neck cancer patients [3]. But it was proved to have low potency and poor bioavailability due to the stoichiometric nature of its action [4]. What is more, side effects of amifostine such as fever, rash, severe nausea, allergy, and acute hypotension have prompted a continuing search for better radioprotector [5–7].

Superoxide radicals produced by ionizing radiation are highly reactive and potentially damaging to cells. The enzyme superoxide dismutase (SOD) neutralizes superoxide radicals by changing it into molecular oxygen and hydrogen peroxide, thereby preventing the formation of highly aggressive compounds such as peroxynitrite. Hydrogen peroxide is then subsequently eliminated by catalase and glutathione peroxidase [8, 9]. SOD is naturally present in human cells and proved to play a key role in cellular defenses against oxidative damage [1]. But as a protein, SOD is too large to freely enter into cells. Although the hypothesis that SOD is radioprotective has been supported by many studies through transgenic experiments [10–15], there were many limitations on its protecting against radiation-induced chronic injury in humans. SOD mimics are another way to overcome the limitation of large molecular weight. Some of them have been proved to be radioprotective in various radiation injury models [16]. But their reaction efficiency of scavenging superoxide anion is still inferior to wild SOD. Their mechanism, selectivity, and toxicity of mimics may vary compared with natural enzyme [16]. In our previous work, we constructed a cell membrane permeable SOD by gene recombinant technique to circumvent this limitation. The recombinant protein was the fusion of hCuZn-SOD (SOD1) and cell-penetrating peptide derived from HIV-1 TAT protein transduction domain TAT (YGRKKRRQRRR). Protein transduction domains are able to carry larger molecules such as oligonucleotides, peptides, full-length proteins, 40 nm iron nanoparticles, bacteriophages, and even 200 nm liposomes across cellular membranes and have proven useful in delivering biologically active cargoes in both in vitro and in vivo models [17–22]. The recombinant SOD had been purified, crystallized, and proved to be effective in preventing and treating the damage of guinea pigs skin caused by single dose UVB radiation [23–25]. What is more, it was proved to be effective in preventing radiation-induced lung injury in mice [26].

Cell permeable SOD was confirmed to have remarkable radioprotective effects compared with wild SOD by above experiments [23, 24, 26]. However, superoxide radicals were not the only harmful reactive chemical species produced by ionizing radiation. To find out whether a cell permeable recombination of different antioxidase would encourage a better outcome, a bifunctional recombinant protein fused with glutathione S-transferase (GST) and cell permeable SOD was constructed firstly and named GST-TAT-SOD. GST is an enzyme that aids in detoxification by speeding up the linking of toxic compounds with glutathione (GSH), thus forming a less reactive substance. Besides that, fusional GST could enhance the expression quantity of soluble bifunctional antioxidase and simplify its purification.

Current study investigated the selective radioprotective effects of this cell permeable bifunctional antioxidant enzyme compared with SOD-TAT and amifostine.

## 2. Materials and Methods

### 2.1. Enzyme and Chemicals

*E. coli* strains with recombinant plasmid of GST-TAT-SOD containing GST, TAT-PTD, and human Cu/Zn-SOD and recombinant protein SOD-TAT were obtained from Institute of Biotechnology, Fuzhou University (Fujian, China). Wild Cu/Zn-SOD was purchased from Datian Huacan Biotechnology Co. Ltd. (Fujian, China). GST affinity chromatography was purchased from Weishi-Bohui Chromatogram Technology Co. Ltd. (Beijing, China). Amifostine was purchased from Meiluo Yinhe Pharmacy Co. Ltd. (Hunan, China). Malondialdehyde (MDA), SOD, catalase (CAT), total antioxidation capacity (T-AOC), and glutathione peroxidase (GSH-Px) reagent kits were purchased from Nanjing Jiancheng Bioengineering Co. Ltd. (Jiangsu, China). Micro-BCA*™* Protein Assay Kit was purchased from Thermo Scientific (USA). RPMI-1640 and fetal bovine serum were purchased from HyClone and Gibco (USA), respectively. All other chemicals were of analytical purity.

### 2.2. Cell Cultures

Normal human liver cell line L-02 cells and human hepatoma cell line Hep G2 cells are available from Shanghai Institute of Biochemistry and Cell Biology (SIBCB). Cells were cultured in RPMI-1640 (HyClone), supplemented with 10% fetal bovine serum (Gibco), 100 U/mL penicillin, and 100 mg/mL streptomycin (Gibco) at 37°C in a 5% CO_2_ humidified chamber.

### 2.3. Mice

Male Kunming mice (Fujian Medical University) weighing 18–22 g each were used at 6–8 weeks of age for these experiments. All mice were housed in an animal room at 22°C in a 12 h light/12 h dark cycle. All mice were given a standard chow diet and water ad libitum. Animal welfare and experimental procedures were carried out in accordance with the Guide for the Care and Use of Laboratory Animals (Ministry of Science and Technology of China, 2006) and were approved by the Review Committee for the Use of Human or Animal Subjects of Institute of Biotechnology Fuzhou University.

### 2.4. Preparation of Recombination Protein GST-TAT-SOD

Bacteria were washed in PBS and lysed in buffer A (10 mM Na_2_HPO_4_, 1.8 mM KH_2_PO_4_, and 2.7 mM KCl, pH 7.3) using a cell disrupter (Sonic Solutions Co., vc-750). The supernatant (extract) was collected after centrifugation of the lysates for 20 min at 12,000 rpm in a HITACHI RX series Himac CF15RX rotor at 4°C. The supernatant containing GST-TAT-SOD was precipitated by incubation with 56.1 g ammonium sulfate per 100 mL of culture medium at 4°C. For purification of GST-TAT-SOD, the precipitate was resuspended in 20 mM PBS, pH 7.4, and purified by GST affinity chromatography resin (Weishi-Bohui Chromtotech Co., Beijing, China) according to the manufacturer's instructions. After binding and washing, the bound protein was eluted with 50 mM Tris-HC1, containing 0.01 M glutathione, pH 8.0. The concentration, SOD activity, and GST activity of the purified protein were determined by BCA Protein Assay Kit (Thermo, USA) and SOD and GST reagent kits (Jiangsu, China), respectively. The SOD and GST activity of purified GST-TAT-SOD were 2476 and 766 U/mL, respectively. Purified protein was concentrated and dialyzed for cellular experiments.

### 2.5. Transduction of Recombination Protein GST-TAT-SOD In Vitro

L-02 cells plated in a 24-well plate were treated with GST-TAT-SOD or wild SOD, then harvested, washed, and lysed using PBS containing 0.5% Triton X-100. Then the cell lysate was removed and centrifuged at 4°C at 13,000 rpm for 15 min, and the SOD activity and the protein content in the supernatant were analyzed spectrophotometrically using SOD diagnostic reagent kits (Nanjing Jiancheng Bioengineering) and BCA Protein Assay Kit (Thermo, USA) following manufacturer's directions, respectively.

In addition, the fusion protein GST-TAT-SOD is labeled using fluorescein isothiocyanate (FITC) following standard methods. Briefly, 2 mg/mL GST-TAT-SOD dialyzed against sodium carbonate was incubated with 40 *μ*g/mL FITC for 1 h at room temperature. Then, labeled proteins are purified from free dye using a gel filtration column.

FITC-labeled GST-TAT-SOD internalized into culture cells was observed under fluorescence microscopy. L-02 cells were seeded into 24-well plates (Greiner, Germany) at a density of 4 × 10^5^ cells per well and cultivated to semiconfluence in RPMI-1640 medium in a humid atmosphere supplemented with 5% CO_2_ for 24 h at 37°C. The culture medium was removed and the cells were washed with PBS twice. Appropriate amount of FITC-labeled GST-TAT-SOD was mixed with fresh medium without serum and then added to the cells and incubated for 3 h with 5% CO_2_ at 37°C. The cells were further washed 5 times with PBS; then the fluorescence was observed under fluorescence microscopy (ZEISS AXIOSKOP-50, Germany).

For quantification of the transduction of GST-TAT-SOD, cells from the different groups were lysed using PBS containing 0.5% Triton X-100. The cell lysate was removed and centrifuged at 4°C at 13,000 rpm for 15 min, and the fluorescence in the supernatant was read with a Synergy H4 Hybrid Multi-Mode Microplate Reader (BioTek Instruments, Winooski, VT). To normalize fluorescence per cell, protein was determined by BCA Protein Assay Kit (Thermo, USA) following manufacturer's directions. Fluorescence was corrected for background signal and normalized for protein content and expressed as fluorescence/*μ*g of protein.

### 2.6. Transduction Experiment In Vivo

Male Kunming mice were randomly divided into 3 groups (*n* = 5 mice per group). The first group (CON) was untreated. The second group (SOD) was injected with 0.5 mL of FITC-labeled SOD (2 kU/mL**)** intraperitoneally and the other group (GST-TAT-SOD) received intraperitoneal injection of 0.5 mL of the FITC-labeled recombinant protein GST-TAT-SOD (2 kU/mL). Three hours after injection, all animals were sacrificed by cervical dislocation. The liver, spleen, lung, brain, and kidney were weighed and 10% homogenates were prepared with ice-cold saline using a homogenizer, respectively. The fluorescence of tissue homogenates from organs was determined with a Synergy H4 Hybrid Multi-Mode Microplate Reader (BioTek Instruments, Winooski, VT). To normalize fluorescence, the protein contents of the 10% homogenates were determined by BCA Protein Assay Kit (Thermo, USA) following manufacturer's directions. Fluorescence was corrected for background signal and normalized for protein content and expressed as fluorescence/*μ*g of protein.

### 2.7. Cytotoxicity: MTT

MTT, a tetrazolium salt, is reduced by viable mitochondria to formazan, which causes a colorimetric change that can be measured at 570 nm. Approximately 1 × 10^5^ cells were seeded per well in 96-well plates and incubated for 48 h in fresh medium along with the protein at the final concentration indicated (three replicates each). Cell viability was estimated by a colorimetric assay using MTT (3-(4,5-dimethylthiazol-2-yl)-2,-5-diphenyltetrazolium bromide) (Sigma Chemical Co.).

### 2.8. Irradiation

Cells were irradiated with X-ray generated by a Linac (IEC 61217) with a nominal potential of 6 MV and a dose rate of 300 UM/min at room temperature.

### 2.9. Clonogenic Tests on Cells following X-Ray Treatment

Confluent 75 cm^2^ flasks of cells were treated with protein or amifostine and irradiated with 2 Gy X-ray subsequently. The dose was confirmed to cause about half percent of surviving fraction of normal cell through an unpublished pretest on the surviving fraction of cells at radiation dose range from 0 to 8 Gy. After irradiation, the cells were trypsinized, counted with a hemocytometer, and diluted in complete media to obtain 100 cells/mL. One milliliter of cell suspension was plated in each well of a 6-well tissue culture plate to obtain 100 cells per well. For each cell line used, experiments were performed in triplicate, and clonogenic tests were performed on following different cells groups. The first group (CON) did not receive any irradiation and was untreated, the second group (XRT) received irradiation only, the third group (XRT+SOD) received irradiation pretreated with wild SOD (250–6000 U/mL) for 3 h, the fourth group (XRT+AMFT) received irradiation pretreated with amifostine (4 *μ*g/mL) for 0.5 h, the fifth group (XRT+GST-TAT-SOD) received irradiation pretreated with GST-TAT-SOD (250–6000 U/mL) for 3 h, and the other groups (XRT+SOD-TAT) received irradiation pretreated with SOD-TAT (250–6000 U/mL) for 3 h. The incubation time of protein was determined according to its transduction effect. The incubation time of amifostine was determined according to its medication guides. Colonies were stained with crystal violet after 14 days and those containing at least 50 cells were counted as surviving colonies. The plating efficiency (PE) and the survival fraction (SF), for each cell line after each treatment, were calculated according to the method proposed by Franken et al. [27]. Protective rate of pretreated groups was calculated as the following formula:

(1) Protective  rate %=SFtreatment−SFXRT100−SFXRT×100.

### 2.10. Measurement of T-AOC, SOD, CAT, MDA, and GSH-PX Activity

L-02 cells and Hep G2 cells in logarithmic growth phase were trypsinized and counted with a hemocytometer. Approximately 3 × 10^6^ cells were seeded into 75 cm^2^ flasks. For each cell line used, experiments were performed in triplicate, and tests were performed on following different cells groups. The first group (CON) did not receive any irradiation and was untreated, the second group (XRT) received 4 Gy X-ray irradiation only, the third group (XRT+SOD) received irradiation pretreated with wild SOD (6000 U/mL) for 3 h, the fourth group (XRT+AMFT) received irradiation pretreated with amifostine (4 *μ*g/mL) for 0.5 h, the fifth group (XRT+GST-TAT-SOD) received irradiation pretreated with GST-TAT-SOD (2000 U/mL) for 3 h, and the other groups (XRT+SOD-TAT) received irradiation pretreated with SOD-TAT (6000 U/mL) for 3 h.

After irradiation, cells were further cultured for 24 h at 37°C. Then, the cells were lysed using PBS containing 0.5% Triton X-100. The cell lysate was removed and centrifuged at 4°C at 13,000 rpm for 15 min, and the activities of SOD (U/mg protein), MDA (nmol/mg protein), CAT (U/mg protein), GSH-PX (U/mg protein), GST (U/mg protein), and T-AOC (U/mg protein) in the supernatant were determined spectrophotometrically using their corresponding diagnostic reagent kits (Nanjing Jiancheng Bioengineering) according to the manufacturer's instructions. The protein content of the lysate was determined by using BCA Protein Assay Kit (Thermo, USA).

### 2.11. ROS Assays

The production of endogenous oxidative stress by-product was assessed using the conversion of 2′,7′-dichlorodihydrofluorescein diacetate (DCHF-DA, Sigma) [28]. Cells were plated in triplicate, grouped as described in Section 2.10, pretreated with protein or amifostine, and incubated with 20 *μ*M DCFH-DA for 1 h in PBS at 37°C. After incubation, the cells were rinsed in PBS and irradiated at a dose of 4 Gy. The fluorescence intensity of irradiated samples was observed within 10 min using a fluorescent microscopy (ZEISS AXIOSKOP-50, Germany) equipped with a digital charge-coupled device camera and a PC for data acquisition and analysis.

### 2.12. Apoptosis Assays

Apoptotic cells were detected using a FITC Annexin-V apoptosis detection kit 1 (BD Pharmingen, San Diego, CA, USA). Briefly, cells (grouped as described in Section 2.10) pretreated with protein or amifostine were irradiated at a dose of 4 Gy and cultured for 24 h at 37°C. Then, the cells were trypsinized and washed with PBS. The washed cells were resuspended in Annexin-V binding buffer containing 10 mM HEPES/NaOH, pH 7.4, 140 mM NaCl, and 2.5 mM CaCl_2_ according to the manufacturer's protocol. The cells were stained simultaneously with FITC-conjugated Annexin-V and PI at room temperature for 15 min in the dark, prior to the addition of binding buffer. The apoptotic cells were measured using a FACScan flow cytometer. The cells were sorted into intact cells (Annexin-V^−^ PI^−^), early apoptotic cells (Annexin-V^+^ PI^−^), late apoptotic cells (Annexin-V^+^ PI^+^), and necrotic cells (Annexin-V^−^ PI^+^).

### 2.13. Statistical Analyses

Statistical analysis of all data was performed using Excel. The results are reported as means ± SE or SEM. The *P* values were determined using the Student two-tailed *t*-test, and *P* < 0.05 or *P* < 0.01 was considered statistically significant.

## 3. Result

### 3.1. Transduction of Recombination Protein GST-TAT-SOD In Vitro and In Vivo

**Figure 1:**
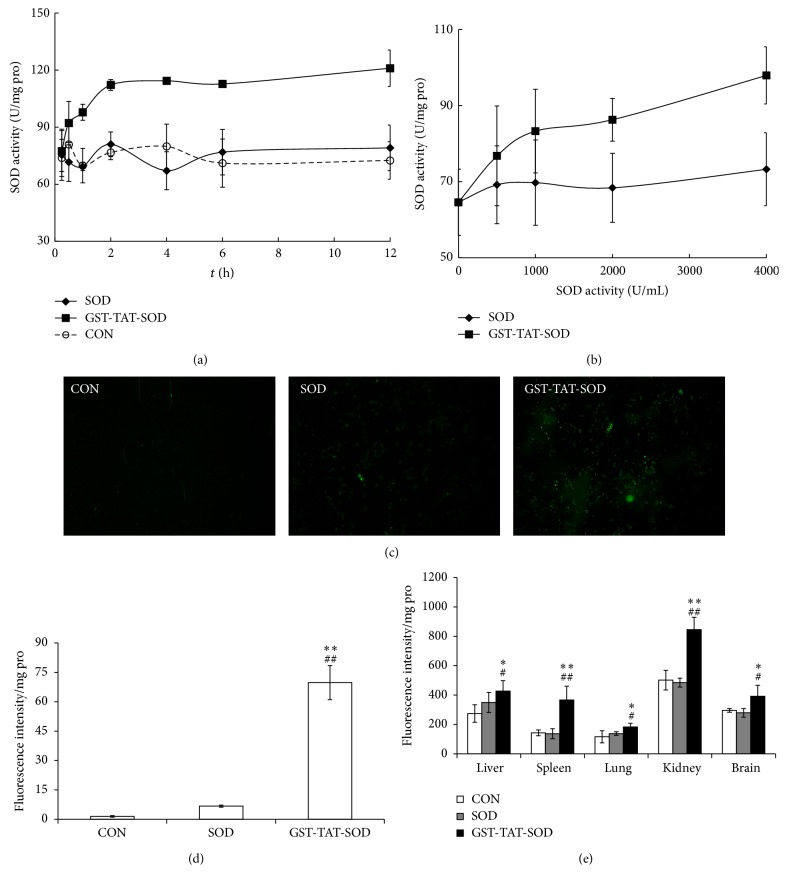
Transduction of GST-TAT-SOD in vitro and in vivo. The transduction activity of the protein was analyzed by measuring the level of the transduced proteins in the cells by SOD activity (a and b) or visualization and quantification of the protein by FITC-labeled (c, d, and e). (a) Protein was added to culture media and incubated for various time intervals. (b) Various concentrations of protein were added to culture media and incubated for 3 h. (c) Visualization of GST-TAT-SOD transduced into cells. L-02 cells were treated with 2 kU/mL FITC-labeled GST-TAT-SOD fusion proteins and control FITC-labeled SOD for 3 h, and the transduced proteins were identified by fluorescence microscopy. (d) Quantification of the transduction of GST-TAT-SOD into cells. L-02 cells plated in a 24-well plate were treated with FITC-labeled protein, then harvested, and washed. The transduction activity of each protein was analyzed by measuring fluorescence intensity in the cells. The bars indicate the means ± SD (*n* = 3, compared with control group, ^##^ *P* < 0.01, compared with wild SOD group, ^*∗∗*^ *P* < 0.01). (e) Transduction of GST-TAT-SOD in vivo. FITC-labeled GST-TAT-SOD or SOD (0.5 mL, 2 kU/mL) was injected intraperitoneally into mice. The homogenates of the liver, spleen, lung, brain, and kidney were prepared and transduction efficiencies were analyzed by measuring fluorescence intensity of tissue homogenates from organs after 3 h. The bars indicate the means ± SD (*n* = 5, compared with control group, ^#^ *P* < 0.05, ^##^ *P* < 0.01, compared with wild SOD group, ^*∗*^ *P* < 0.05, ^*∗∗*^ *P* < 0.01).

The restoration of enzymatic activities of transduced fusion protein into cells is critical for the application of protein transduction technology or therapeutic use. Therefore, we determined the SOD activities of cells transduced with GST-TAT-SOD firstly. As shown in Figure 1(a), GST-TAT-SOD was successfully delivered into cells, whereas wild SOD was not. The enzyme activity of SOD in transduced cells increased rapidly in 2 h and basically levelled off in the next 10 hours (Figure 1(a)). Besides that, the amount of transduced fusion protein also increased in a dose-dependent manner (Figure 1(b)). To visualize the transduction of GST-TAT-SOD, fusion protein was labeled by FITC, internalized into culture cells, observed under fluorescence microscopy, and quantified by fluorescence Microplate Reader. As shown in Figures 1(c) and 1(d), compared with wild SOD, GST-TAT-SOD (2 kU/mL) was efficiently transduced into cells after incubation for 3 h (*P* < 0.01). In vivo, GST-TAT-SOD exhibited excellent transduced ability as was shown in Figure 1(e). The fluorescence intensity in liver, lung, spleen, kidney, and brain tissues of mice almost had no difference between CON group and wild SOD group, while the fluorescence intensity in those tissues of mice treated with GST-TAT-SOD was considerably increased compared to other two groups (*P* < 0.05; *P* < 0.01). This result implicates that GST-TAT-SOD could be effectively transduced into different organs in vivo.

### 3.2. In Vitro Cytotoxicity

**Figure 2:**
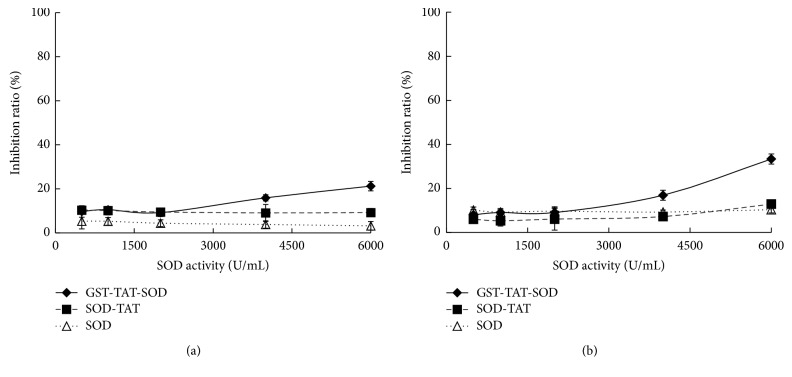
Cytotoxic effects of GST-TAT-SOD on cells. Approximately 1 × 10^5^ cells were seeded per well in 96-well plates and incubated for 48 h in fresh medium along with the protein at the final concentration indicated (three replicates each). The bars indicate the means ± SD (*n* = 3). (a) L-02 cells. (b) Hep G2 cells.

The MTT assay was used to assess the effect of GST-TAT-SOD concentration on cell viability in L-02 and Hep G2 cells as shown in Figure 2. GST-TAT-SOD (500–2000 U/mL) had little cytotoxicity on both cells. Higher concentration GST-TAT-SOD (6000 U/mL) resulted in inhibition of L-02 cell proliferation about 21.25% (Figure 2(a)). A dose-dependent (2000–6000 U/mL) reduction in cell viability of GST-TAT-SOD was observed in Hep G2 cell lines, up to about 33.39% (Figure 2(b)). SOD-TAT and wild SOD both were nontoxic to two cell lines (Figure 2).

### 3.3. Clonogenic Tests on Cells following X-Ray Treatment

**Figure 3:**
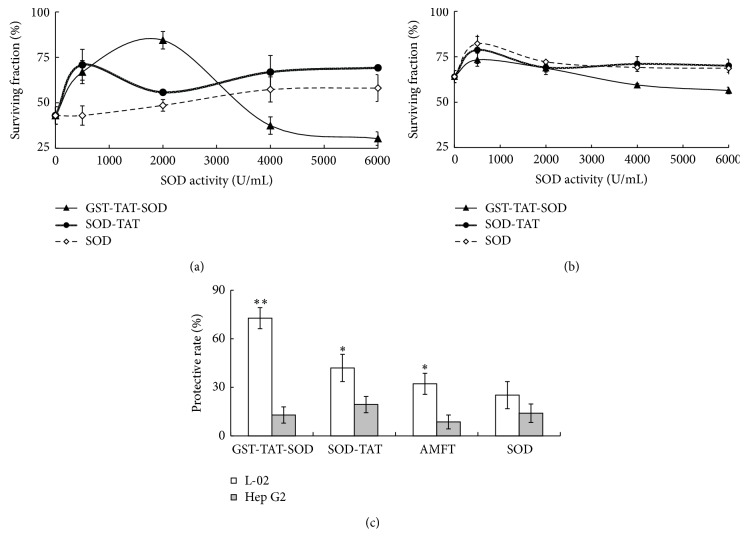
Clonogenic tests on L-02 (a) and Hep G2 (b) cells following X-ray treatment. The cell strains were treated in the presence of protein or amifostine and irradiated with 2 Gy X-ray subsequently. After irradiation, the cells were detached with trypsin-EDTA and plated at a concentration of 100 cells/dish in drug-free medium. Fourteen days later the colonies were fixed and counted by using a crystal violet staining to evaluate the number of colonies present in the dishes. (c) Comparison of protective rate between three proteins and amifostine. The bars indicate the means ± SD (*n* = 3, compared with group received irradiation only, ^*∗*^ *P* < 0.05, ^*∗∗*^ *P* < 0.01).

Colony-forming assays were used to determine the radioprotective effect of cell permeable antioxidant enzyme (Figure 3). As shown in Figure 3(a), surviving fraction of GST-TAT-SOD on L-02 cells colonies tended to follow a bell curve over protein concentration. The effect of SOD-TAT represented a little bell curve in low concentration (<2000 U/mL), while higher concentration showed upward trend in concentration. Compared with two cell permeable SOD, wild SOD showed a most slowly rising trend in concentration. Otherwise, all of three proteins in low concentration (<500 U/mL) seemed to enhance the surviving fraction of Hep G2 cells to a certain degree. In higher concentration, GST-TAT-SOD showed a continuous downward trend, while SOD-TAT and wild SOD tend to level off (Figure 3(b)). When protective effects on L-02 and Hep G2 cells are considered together, the optimal doses of GST-TAT-SOD, SOD-TAT, and wild SOD were 2000 U/mL, 6000 U/mL, and 6000 U/mL, respectively. The optimal dose of radioprotector used in clinic amifostine was proved to be 4 *μ*g/mL (unpublished). As was shown in Figure 3(c), amifostine, SOD-TAT, and GST-TAT-SOD all had significant protective rate on irradiated L-02 cells (*P* < 0.05; *P* < 0.01). GST-TAT-SOD exhibited the optimal effect among above three pretreated groups. Wild SOD have a little protective effect but have no significance. All of them have a certain degree of protective effect on Hep G2 cells but no significant enhancement was observed.

### 3.4. Measurement of SOD, CAT, T-AOC, GSH-PX, and MDA Activity

**Figure 4:**
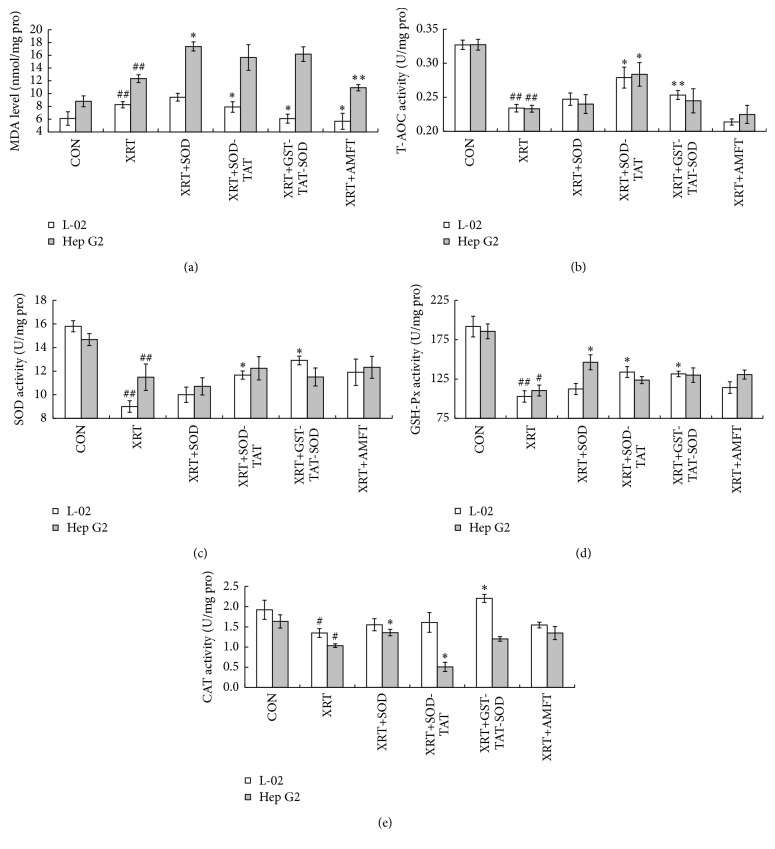
Biochemical estimations of both cells. (a) MDA level; (b) T-AOC activity; (c) SOD activity; (d) GSH-PX activity; (e) CAT activity. Cells were pretreated with protein or amifostine and irradiated with 4 Gy X-ray subsequently. After irradiation, the cells were further cultured for 24 h at 37°C. Then, the cells were lysed and centrifuged, and the antioxidant activities in the supernatant were determined subsequently. The bars indicate the means ± SD (*n* = 3). The XRT group was compared with the control group (^#^ *P* < 0.05, ^##^ *P* < 0.01). The pretreated group was compared with the XRT group (^*∗*^ *P* < 0.05, ^*∗∗*^ *P* < 0.01).

A significant reduction in the activity of antioxidant enzymes (SOD, CAT, and GSH-PX) and a remarkable increase in the level of MDA were observed in both L-02 and Hep G2 XRT groups 24 h after irradiation (Figure 4(a), *P* < 0.05; *P* < 0.01). T-AOC of both cells was also significantly decreased after irradiation (Figure 4(b), *P* < 0.01). Compared with XRT group, pretreatment of amifostine significantly (*P* < 0.05; *P* < 0.01) decreased the level of MDA in both two cell lines but failed to keep the T-AOC activity of cells (Figures 4(a) and 4(b)). This treatment seemed to enhance the SOD activity, GSH-Px activity, and CAT activity of irradiated cells to some extent, but those indexes were not significant in comparison with XRT group (Figures 4(c)–4(e)). Wild SOD pretreatment failed to significantly improve all antioxidant indexes in L-02 cells but it essentially increased the CAT activity, GSH-PX activity, and MDA level in Hep G2 cells (Figures 4(c)–4(e), *P* < 0.05). SOD-TAT pretreatment greatly increased the SOD, GSH-Px, and T-AOC activity and decreased the MDA level in L-02 cells (Figure 4, *P* < 0.05). It had no obvious effect on antioxidant index in Hep G2 cells expect CAT activity and T-AOC activity (Figures 4(e) and 4(b), *P* < 0.05). GST-TAT-SOD treatment which was the best one among four pretreatment groups remarkably kept all antioxidant indexes in L-02, especially CAT activity and MDA level, which reached or were higher than those of the control group (Figures 4(e) and 4(a), *P* < 0.05). Furthermore, GST-TAT-SOD treatment had no noteworthy protective effect on those indexes in Hep G2 cells (Figure 4).

### 3.5. ROS Assay

**Figure 5:**
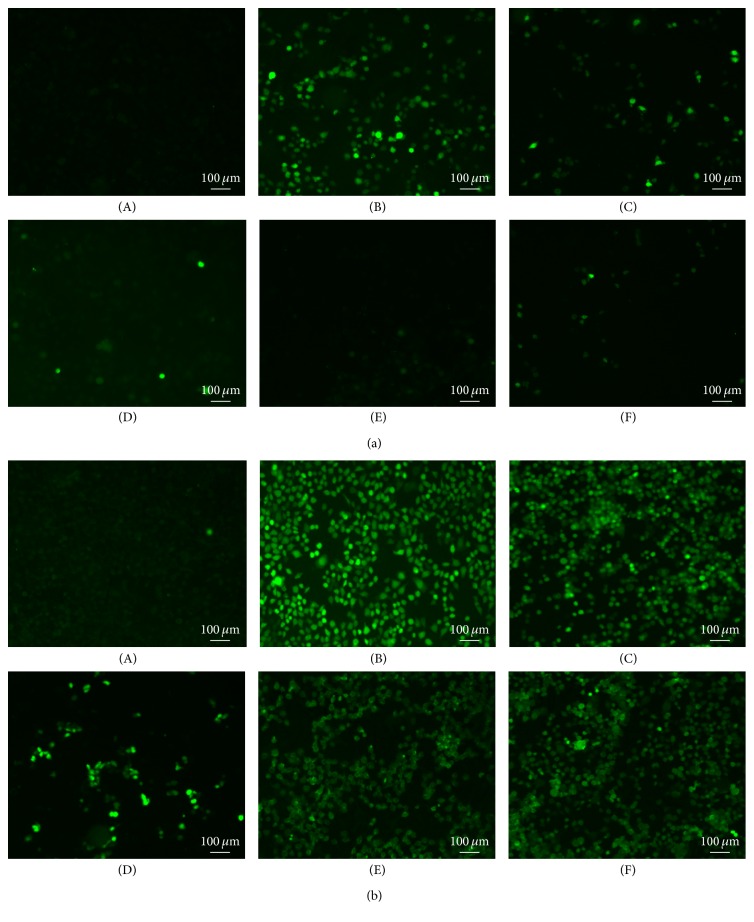
DCFH-DA detection of ROS in L-02 (a) and Hep G2 (b) cells treated with X-ray radiation. Cells were plated in triplicate, pretreated with protein or amifostine, and incubated with DCFH-DA for 1 h in PBS at 37°C. After incubation, the cells were rinsed in PBS and irradiated at a dose of 4 Gy. The fluorescence intensity of irradiated samples was observed within 10 min using a fluorescent microscopy. (A) CON; (B) XRT; (C) XRT+SOD; (D) XRT+AMFT; (E) XRT+GST-TAT-SOD; (F) XRT+SOD-TAT.

ROS production in both two cells induced by X-ray radiation was evaluated using a ROS-dependent oxidation of DCFH-DA. A remarkable increase in ROS production was observed in both two cells after irradiation (Figures 5(a)-(B) and 5(b)-(B)). Pretreatment of L-02 cells with amifostine, SOD-TAT, or GST-TAT-SOD, especially GST-TAT-SOD, significantly suppressed the elevation of ROS production (Figures 5(a)-(D), 5(a)-(E), and 5(a)-(F)). As other cancer cells, Hep G2 cells have higher levels of ROS than normal cells (Figure 5(b)-(A)). After irradiation, higher levels of ROS were induced in Hep G2 cells than that of L-02 cells (Figure 5(b)-(B)). Pretreatment with SOD-TAT or GST-TAT-SOD slightly decreased the elevation of ROS production (Figures 5(b)-(E) and 5(b)-(F)) while amifostine pretreatment significantly suppressed that (Figure 5(b)-(D)). By contrast, wild SOD could not prevent the production of intracellular ROS induced by irradiation (Figures 5(a)-(C) and 5(b)-(C)).

### 3.6. Apoptotic Index

**Figure 6:**
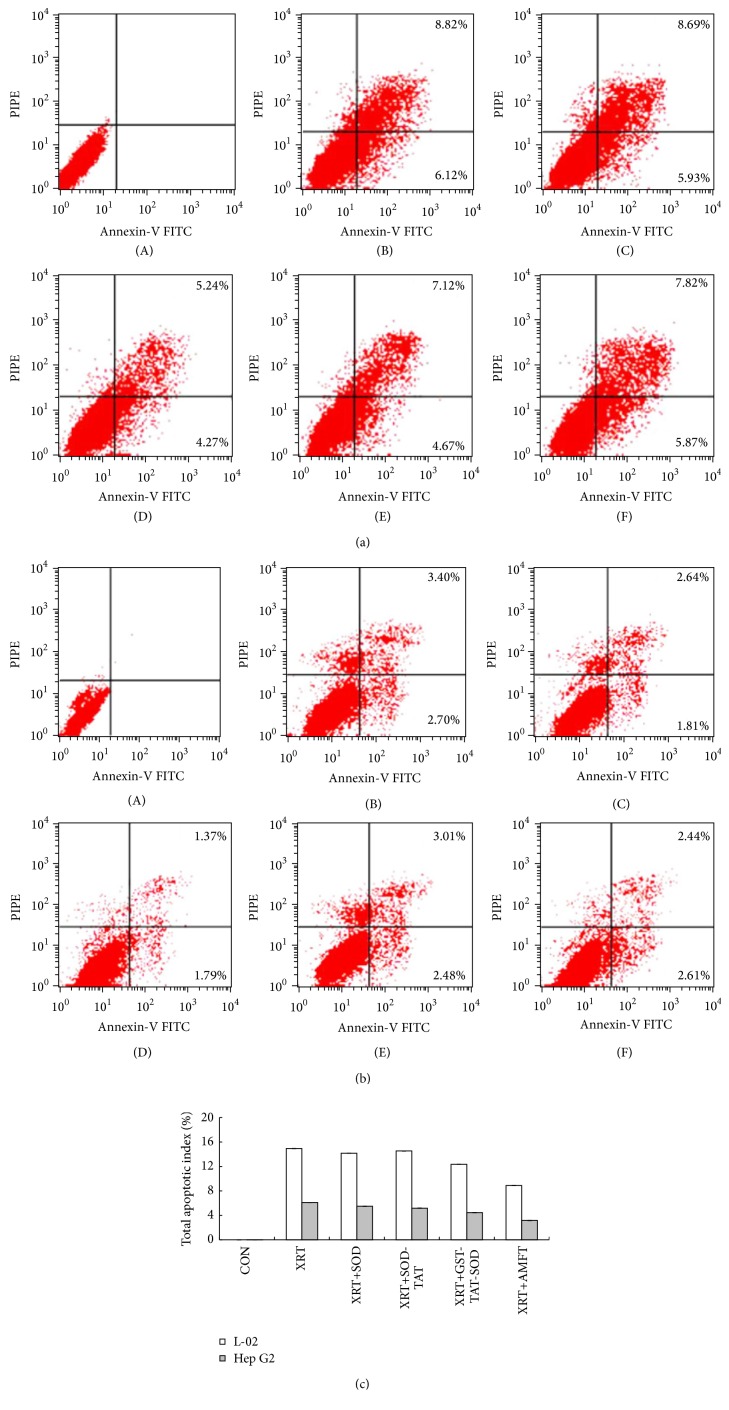
Annexin-V/PI analysis of apoptosis in L-02 (a) and Hep G2 (b) cells at 24 h after treatment with 4 Gy of irradiation. Cells were stained with Annexin-V FITC and PI and detected by flow cytometry. The lower right quadrant (Annexin-V^+^ PI^−^) represents early apoptosis, whereas upper right quadrant (Annexin-V^+^ PI^+^) represents late apoptosis ((A) CON; (B) XRT; (C) XRT+SOD; (D) XRT+AMFT; (E) XRT+GST-TAT-SOD; (F) XRT+SOD-TAT). (c) Total apoptotic index of cells with different treatment.

To further confirm the effect of GST-TAT-SOD on irradiation-induced apoptosis in both L-02 and Hep G2 cells, Annexin-V-FITC/PI staining experiment was performed (Figure 6). The number of Annexin-V-positive cells increased in both L-02 and Hep G2 cells after irradiation treatment; the number of total apoptotic indexes was 14.94% and 6.10%, respectively. Amifostine pretreatment could reduce the apoptotic index of both L-02 and Hep G2 cells to 9.51% and 3.16%, respectively. Pretreatment with wild SOD failed to lower the apoptotic index of L-02 cells while the apoptotic index of Hep G2 cells with same treatment was decreased to 4.45%, respectively. SOD-TAT pretreatment could lessen the apoptotic index of both L-02 and Hep G2 cells to 13.69% and 5.05%, respectively. Pretreatment with GST-TAT-SOD could go down the apoptotic index of two cell lines to 11.79% and 5.49%, respectively.

## 4. Discussion

In this study, a bifunctional protein, GST-TAT-SOD, a recombinant fusion of GST, SOD, and TAT from* E. coli,* was prepared. Firstly, GST-TAT-SOD was proved to be cell permeable and safe to normal cells. Then, the radioprotective effect of bifunctional GST-TAT-SOD on radiation-induced cellular damage was tested in comparison with monofunctional SOD-TAT and amifostine.

Our results show that intracellular ROS were induced in both human normal liver cells and human hepatoma cells by ionizing radiation (Figure 5) which caused a significant reduction of antioxidant system (Figure 4), decline in colony-forming ability, and apoptosis of cells (Figures 3 and 6). Amifostine could remove intracellular ROS in irradiated normal liver cells effectively (Figure 5) and reduce subsequent radiation injury (Figures 3, 4, and 6).

SOD enzymes are indispensable and ubiquitous antioxidant defenses protecting oxygen-utilizing cells from the toxicity of the ROS produced by irradiation. But our results showed that wild SOD had little protective effect for its inefficient delivery across the biological membranes due to the large molecular size (Figures 3, 4, 5, and 6).

Intramuscular injection of liposomal CuZn-SOD, intratracheal injections of Mn-SOD plasmid-liposome or Mn-SOD adenovirus gene therapy, and overexpression of EC-SOD in transgenic mice prior to irradiation were effective in attenuating radiation damage [10, 14, 29–31] which further indicated the importance of intracellular delivery of SOD for the radioprotective effect. But these applications in human are far away from practical due to the apparent technical difficulties.

SOD mimics provide a novel potential development pattern of SOD. Mn porphyrins are most valid SOD mimics up to now and have been proven to be anticancer and radioprotective [16]. But it still needs further improvement to match the catalytic efficiency of natural enzyme. Otherwise, their action mechanism is varied due to vastly different sterics [16].

SOD fused with cell-penetrating TAT-PTD overcame the above-mentioned shortcoming. Both monofunction and bifunctional cell membrane permeable SOD, SOD-TAT, and GST-TAT-SOD can enter into irradiated normal cells (Figure 1), remarkably clear up intracellular redundant ROS (Figure 5), maintain antioxidant system (Figure 4), enhance colony-forming ability (Figure 3), and suppress apoptosis (Figure 6).

Bifunctional GST-TAT-SOD was superior to monofunctional SOD-TAT in many ways such as higher antioxidant capacity, colony-forming ability, and lower apoptosis index of irradiated normal cells which may be due to the additional fusion of GST. Via a sulfhydryl group, GSTs catalyze the conjugation of reduced glutathione (GSH) to electrophilic centres on a wide variety of substrates [32]. This activity detoxifies endogenous compounds such as peroxidised lipids [33]. Although GST activity of irradiated cells would not be impacted with low irradiation dose [34], increasing intracellular GST activity did help to enhance the ability to remove free radicals and antioxidant capacity of GST-TAT-SOD compared to that of SOD-TAT (Figures 5 and 4). As such, the radioprotective effect of bifunctional antioxidant enzymes in low dose (2000 U/mL) was better than that of single-function antioxidant enzymes in higher dose (6000 U/mL).

One of the major concerns with the use of radioprotector during the course of radiotherapy is the possibility of tumor protection. Many antioxidants such as alpha tocopherol and beta carotene used during the course of radiotherapy were associated with evidence of poorer tumor control in randomized trials [35, 36], although they could reduce normal tissue toxicity in many instances with promising results.

Amifostine has been shown to concentrate more rapidly in normal tissues than in tumor tissues in studies of tumor-bearing animals [37]. But concerns about tumor protection and toxicity of this agent have led to controversy regarding the appropriate setting for its use [38]. Our results showed that although amifostine was a good radioprotector of normal hepatocytes, it showed some degree of protective effects on hepatoma cells at the same time (Figures 3, 4, 5, and 6). Instead, both SOD-TAT and GST-TAT-SOD seemed to eliminate free radical cell selectively (Figure 5(b)) which may be due to their selective cell-penetrating capability on cell lines [39]. Particularly the latter seemed to have no significant effect on antioxidant system (Figure 4), SF (Figure 3(c)), and apoptotic index of irradiated hepatoma cells (Figure 6). Otherwise, intraperitoneal injection of GST-TAT-SOD was proved to be successfully transduced into different organs in mice, including the brain where amifostine could not reach (Figure 1(e)). What is more, protein fusion with TAT could be delivered by a variety of routes, including oral administration [40] and parenteral administration such as transdermal administration [23, 24] and intraperitoneal injection [26, 41]. All these showed that bifunctional GST-TAT-SOD has the potential to be a safer and more efficient radioprotector for clinical applications in radiotherapy or intentional exposures to ionizing radiation. More detailed selectively protective mechanism and protective effect in vivo of GST-TAT-SOD need to be studied in the future for a comprehensive assessment of its potential. However, fusion of two antioxidant enzymes and cell-penetrating peptides is potentially valuable in the development of radioprotective agent.

## 5. Conclusions

The present study has not yet provided various evidences for the selectively radioprotective effect of bifunctional GST-TAT-SOD on irradiated cells. Nonetheless, it confirms that GST-TAT-SOD pretreatment is safe and better than amifostine or SOD-TAT pretreatment to selectively scavenge intracellular free radical, maintain antioxidant system, enhance colony-forming ability, and suppress apoptosis.
